# Arterial Spin Labelling Perfusion Changes in Persistent Migrainous Aura Without Infarction

**DOI:** 10.7759/cureus.88129

**Published:** 2025-07-16

**Authors:** Bhadra Sajeev Nair, Vandana Sudheer, Boby Varkey Maramattom

**Affiliations:** 1 Medicine, Malankara Orthodox Syrian Church Medical College Hospital, Kolenchery, Ernakulam, IND; 2 Department of Neurology, Lourdes Hospital, Kochi, IND; 3 Department of Radiology, Lourdes Hospital, Kochi, IND

**Keywords:** arterial spin labelling (asl), cortical spreading depolarization, migraine disorder, scintillating scotoma, visual aura

## Abstract

Migraine is a common disorder characterized by recurrent, usually unilateral, pulsatile headaches often accompanied by nausea, vomiting, photophobia, phonophobia, and fatigue. Visual aura is the most common of all aura symptoms in migraine patients. The aura and headache phases of migraine are caused by intracranial vasoconstriction and extracranial vasodilation, respectively. Migrainous aura without infarction refers to migraine aura symptoms that persist for an extended period of one week or longer without any evidence of infarction on imaging. Arterial spin labelling (ASL) is a non-invasive magnetic resonance imaging technique used to quantitatively assess cerebral blood flow. It can be used to track perfusion changes over the migraine cycle. This case report highlights the utility of ASL as a non-invasive tool to detect reversible perfusion changes in persistent migrainous aura, aiding in differentiating it from ischemic events, offering insights into underlying pathophysiology, such as cortical spreading depression, and informing more accurate clinical management.

## Introduction

Migraine is a prevalent neurological disorder characterized by recurrent, often unilateral headaches with a pulsatile quality, frequently accompanied by nausea, vomiting, generalized fatigue, and photophobia. As of 2025, the prevalence of persistent migraine without aura in India is estimated to be 10%-15%, with visual aura being the most common type (over 90% of aura cases) [1]. Scintillating or fortification scotoma, characterized by visual blurring, distortions, flickering, and shimmering, is most frequently observed. A primary dysfunction in central neuronal excitability is thought to underlie both aura and headache phases, with a propagating wave of reduced cerebral perfusion originating in the occipital cortex and advancing anteriorly. It resembles cortical spreading depolarization (CSD), wherein transient neuronal and glial depolarization suppresses neural activity and diminishes blood flow. This extent of hypoperfusion is sufficient to cause ischemic symptoms. Prolonged migrainous aura indicates prolonged cerebral hypoperfusion and may progress on to infarction. As the headache phase sets in, there is a relative hyperperfusion. Demographic trends show that migraines with aura most often affect females. Magnetic resonance imaging (MRI) helps rule out stroke mimics like transient ischemic attacks, seizure, reversible cerebral vasoconstriction syndrome, and intracranial hemorrhage. Arterial spin labelling (ASL) is a non-invasive MRI technique that quantitatively assesses cerebral blood flow that enables recognition of hypoperfusion or hyperperfusion states. ASL can be used to track perfusion changes over the migraine cycle. According to a 2024 study conducted among 1155 migraine patients, ASL done during the ictal phase showed hypoperfusion throughout aura symptoms, followed by hyperperfusion [2]. Herein, we present the case of a 32-year-old woman with chronic headache associated with persistent migrainous visual aura without infarction on MRI.

## Case presentation

**Figure 1:**
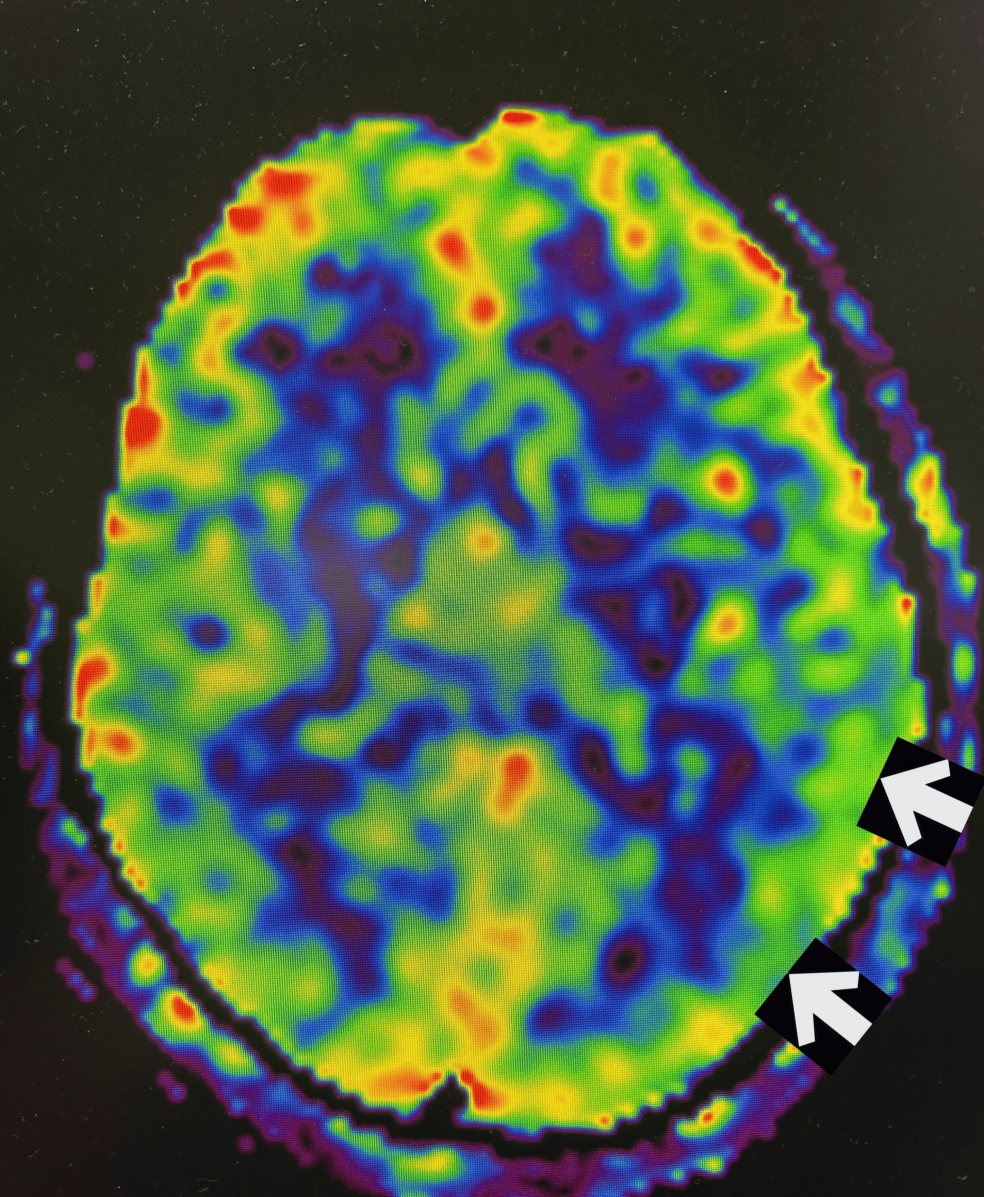
Arterial spin labelling imaging shows hypoperfusion of the left parieto-occipital cortex during the persistent aura phase (arrows)

A 32-year-old female patient presented with a continuous flickering visual aura localized to the right upper quadrant of her visual field of the right eye, described as “seeing fireflies” for one week. The patient, however, did not experience headache. She had a history of chronic migraine, with headaches on more than 15 days every month for over three months, with aura. The patient did not report any regular medication, sensory changes, or menstrual abnormalities. Blood investigations, including complete blood count, kidney function tests, liver function test, lipid profile, blood glucose levels, thyroid profiles, and viral serology, were negative. Electroencephalogram (EEG) did not show epileptiform discharges. Arterial spin labelling during the ictal phase showed hypoperfusion of the left-sided parieto-occipital cortex as evidenced by asymmetry in signal (Figure 1). She was managed with beta-antagonists (oral propranolol 40 mg) and selective serotonin reuptake inhibitors (oral lasmiditan 50 mg), and her symptoms improved.

## Discussion

**Figure 2:**
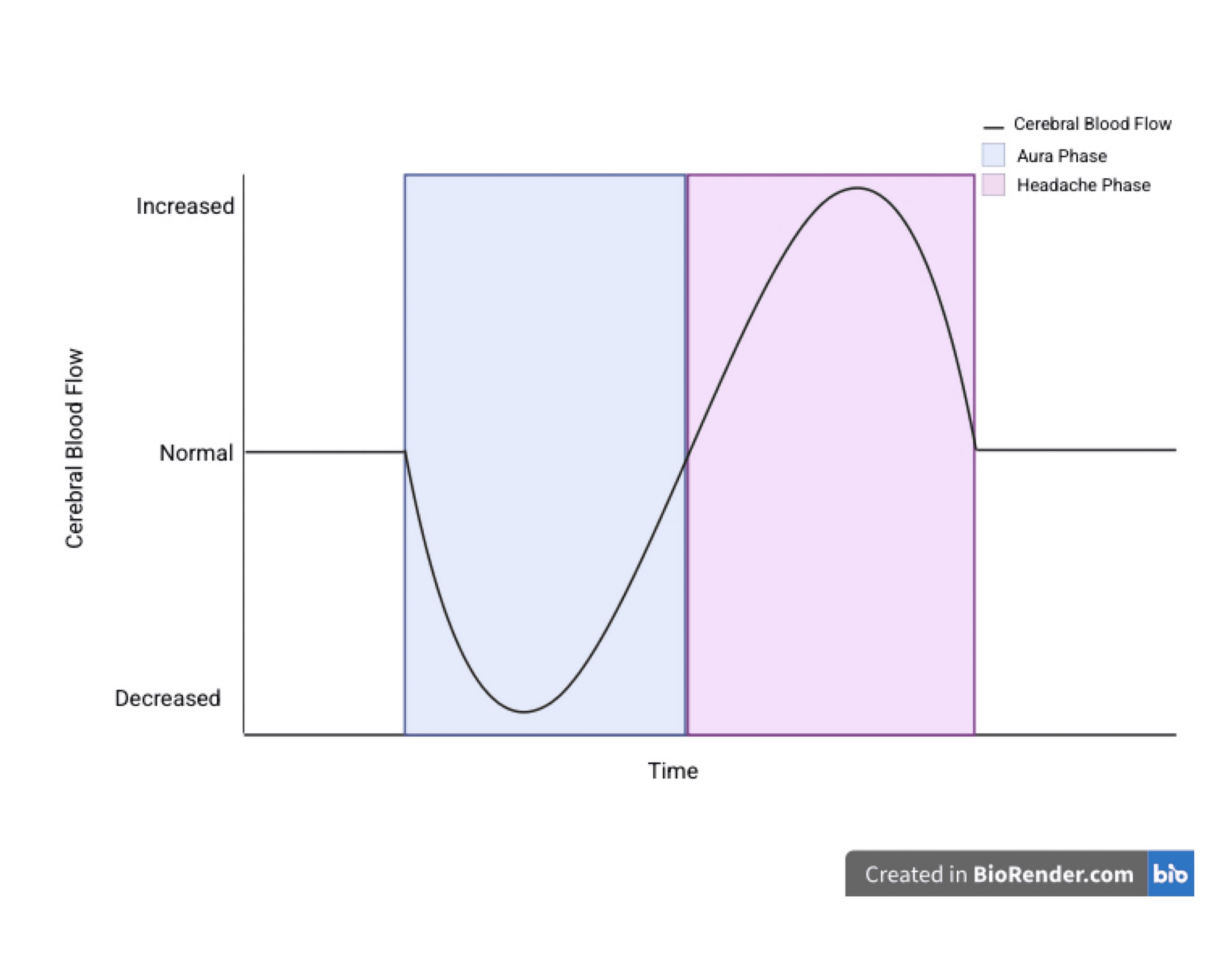
Time course of changes in cerebral blood flow in the migraine cycle Image credits: Created by authors on BioRender software (BioRender, Toronto)

Persistent migrainous aura without infarction (PMA) is a rare complication of migraine with aura, in which typical aura symptoms such as visual disturbances, sensory changes, or speech difficulties persist for one week or longer without evidence of brain infarction on neuroimaging [1]. Migraine begins with intracranial vasoconstriction, leading to decreased blood flow in the occipital cortex, causing aura symptoms through a process known as cortical spreading depolarization (Figure 2). During CSD, endogenous calcitonin-gene related peptide (CGRP) is released from cortical tissue in a calcium-dependent manner, contributing to secondary vasodilation and neurogenic inflammation. However, this vasodilation is localized and insufficient to fully counteract the widespread vasoconstrictive response initiated by CSD. This results in transient neuronal inhibition and can mimic ischemia. If the aura is prolonged, it may lead to sustained hypoperfusion and potentially cerebral infarction. The headache phase follows, marked by extracranial vasodilation and relative cerebral hyperperfusion [3,4].

This 32-year-old female patient presented to the outpatient department with isolated visual aura, that is, scintillating scotoma, persisting for one week in the absence of headache. A classical aura typically evolves and traverses the visual field, and recent studies suggest that approximately 20% of migraine patients may exhibit transient neurological symptoms without any accompanying headache. In a systematic case-based review article including 47 PMA cases, it was rightly pointed out that “accurate history taking is the most important diagnostic tool”. Clinical examinations and investigations in PMA would usually be normal [5,6].

Generally, auras without headache do not require treatment. The most effective drug in the treatment of PMA is lamotrigine [5,6]. An inhaled beta agonist, isoproterenol, may shorten the aura. Aura is now believed to result from neuronal depression, not ischemia, making vasodilators and triptans ineffective or harmful if used during this phase. Triptans, especially in oral forms, do not abort aura and may not prevent the headache even when injected. Calcium channel blockers, especially verapamil, are often very effective. Meclofenamate and naproxen are two rapid-acting non-steroidal anti-inflammatory drugs that are occasionally effective in reducing the duration of symptoms. Antiepileptic drugs such as valproic acid, gabapentin, and topiramate are also effective in reducing the frequency of PMA [7].

## Conclusions

Migraine aura without headache should be considered in individuals experiencing recurrent, transient symptoms, particularly those of visual, neurological, or vestibular nature. While migrainous auras are typically vivid, scintillating, and dynamic, often manifesting as geometric patterns, ischemic visual disturbances are more commonly dark, static, and featureless in appearance. Our patient, who reported with a one-week history of scintillating scotoma, was diagnosed with persistent migrainous aura without infarction with the help of non-invasive ASL-MRI, which revealed hypoperfusion in the left parieto-occipital cortex. ASL perfusion imaging in migrainous aura without infarction reveals a reversible, biphasic blood flow pattern, initial hypoperfusion followed by hyperperfusion, that helps distinguish it from stroke, with PMA being a rare clinical entity.
